# Associations Between Arterial Stiffness and Metabolic Target in Children and Adolescents With Type 1 Diabetes Treated in a Modern Setting

**DOI:** 10.1155/2024/5528717

**Published:** 2024-10-26

**Authors:** Julie A. Damm, Amalie Dalgas-Madsen, Agnes M. K. Bech, Kasper A. Pilgaard, Flemming Pociot, Tine W. Hansen, Jesper Johannesen

**Affiliations:** ^1^Steno Diabetes Center Copenhagen, Herlev, Denmark; ^2^Department of Pediatrics and Adolescent Medicine, Herlev and Gentofte Hospital, Copenhagen University Hospital, Herlev, Denmark; ^3^Department of Clinical Medicine, University of Copenhagen, Copenhagen, Denmark

## Abstract

**Objective:** To investigate the prevalence of elevated arterial stiffness and associations to known and potentially novel risk factors in a modern European technology-based cohort of children and adolescents with type 1 diabetes.

**Research Design and Methods:** Cross-sectional study, including 127 children recruited from Pediatric Diabetes Departments across Eastern Denmark between May 2022 and January 2024. Arterial stiffness was assessed as carotid–femoral pulse-wave-velocity (cfPWV) using the Sphygmocor XCEL system. Unadjusted and adjusted linear regression models explored associations between cfPWV and other risk factors. Adjustments included age, sex, diabetes duration, time-in-range, hemoglobin A1c (HbA1c), body mass index (BMI) *z*-score, low-density lipoprotein (LDL)-cholesterol, and mean arterial pressure (MAP).

**Results:** Median (interquartile range [IQR]) age was 14.2 years (12.0, 16.4), diabetes duration was 4.7 years (2.7, 8.4), HbA1c level was 7.0% (6.5, 7.9), (53 mmol/l: 48–63), time-in-range was 63% (53–75), and 52% were male. The majority were treated with continuous-subcutaneous-insulin-infusion (82%), and all (except two) used continuous-glucose-monitors. The prevalence of elevated arterial stiffness (cfPWV *z*-score over the 90th percentile) was 16%. Unadjusted analyses demonstrated higher cfPWV was associated with longer diabetes duration, higher age, HbA1c, MAP, and liver stiffness, and lower time-in-range and insulin sensitivity. Higher cfPWV remained associated with higher age (standardized *β* (confidence interval (CI) 95%): 0.38 (0.27, 0.48); *p*  < 0.001) and lower time-in-range (−0.15 ((−0.26), (−0.03)); *p*  < 0.011) after adjustment.

**Conclusions:** Despite modern treatment technology and better overall metabolic control, children and adolescents with type 1 diabetes present with a high prevalence of elevated arterial stiffness. Higher arterial stiffness was associated with higher age and lower time-in-range, independent of other risk factors, including HbA1c.

## 1. Introduction

Increased arterial stiffness is present already in children and adolescents with type 1 diabetes compared to healthy controls [1–3]. Higher arterial stiffness is an independent risk factor for cardiovascular complications and mortality in various populations [4], including adults with type 1 diabetes [5].

Increased arterial stiffness is considered an early sign of cardiovascular disease as it is often present long before clinical signs of vascular disease emerge [6]. Carotid–femoral pulse-wave-velocity (cfPWV) is considered the gold standard of noninvasive measure of arterial stiffness in both children and adults [7], with increased cfPWV indicating reduced vessel elasticity and compliance.

In adults with type 1 diabetes, higher cfPWV is associated with longer diabetes duration, higher age, systolic blood pressure, hemoglobin A1c (HbA1c), and the presence of obesity [6]. In children and adolescence with type 1 diabetes, cross-sectional baseline data from the large SEARCH study demonstrated an association between increased cfPWV and higher mean arterial pressure (MAP) as well as central obesity [1, 8]. Follow-up assessment after 5 years demonstrated lower insulin sensitivity at baseline [9], a higher increase in waist circumference, and low-density lipoprotein (LDL)-cholesterol from baseline to follow-up were factors independently associated with higher cfPWV [10]. The SEARCH study also demonstrated that the increase in cfPWV seen in adolescents with type 1 diabetes compared to healthy controls was only partly explained by traditional cardiovascular risk factors [1]. Thus, in children and adolescents with type 1 diabetes, the increase in cfPWV seems to be associated with modifiable and non-modifiable risk factors different from those in adults with type 1 diabetes.

Glycemic control is considered the major determinant of long-term prognosis for people with type 1 diabetes [11]. Only a few studies have investigated the association between glycemic control and cfPWV in children and adolescents with type 1 diabetes, and the findings are diverse. While two large cross-sectional studies (based on the SEARCH study), comprising 535 children [8] and 1376 adolescents with type 1 diabetes [12], respectively, found no association between arterial stiffness and glycemic control when adjusting for sex, age, and diabetes duration, a longitudinal study of 298 children with type 1 diabetes over 5 years demonstrated that increasing HbA1c was associated with higher cfPWV during follow-up [10].

Advancements in blood glucose monitoring and insulin dosing technologies have significantly improved glycemic outcomes in children and adolescents with type 1 diabetes over the past decade [13, 14]. A recent smaller study assessed the association between cfPWV and modern insulin delivery methods in a cross-sectional design [15] and found no association between insulin delivery methods (continuous subcutaneous insulin infusion [CSII] vs. multiple daily injections) and cfPWV, nor between HbA1c levels and cfPWV [15].

To the best of our knowledge, no larger studies have investigated the association between cfPWV and metabolic target in children and adolescents with type 1 diabetes treated in a modern technology-based setting. Thus, this study aims to address this gap by (1) describing the prevalence of elevated arterial stiffness in a modern technology-based European cohort of children and adolescents with type 1 diabetes and by (2) exploring the association between cfPWV as an indicator of arterial stiffness and known as well as potentially novel risk factors.

## 2. Research Design and Methods

### 2.1. Study Design and Population

This cross-sectional study is part of a larger study named “The DetectChild Study,” investigating early signs of diabetes complications in children and adolescents with type 1 diabetes. The present analysis comprises the first 151 children/adolescents included, of whom 127 (84%) participants had a measurement of cfPWV. We excluded one participant with a microvascular complication (retinopathy) and one because of daily smoking; thus, the final analyses included 125 participants.

Children and adolescents aged 6–18 years and diagnosed with type 1 diabetes for more than 12 months were recruited through outpatient clinics at pediatric diabetes departments across the Eastern part of Denmark from May 2022 to January 2024.

All participants were invited for a single study visit at Steno Diabetes Center Copenhagen, Denmark, where all examinations were performed after an overnight fasting (>8 h). If the participants had low blood glucose during nighttime and therefore in need of carbohydrate intake, the visit was rescheduled.

Height and weight were measured without shoes and wearing light clothes to the nearest 0.1 cm and 0.1 kg, respectively, using the fully automated SECA 287. Body mass index (BMI) was calculated as weight (kg)/height (m)^2^. BMI *z*-scores were obtained from a large national cohort [16].

Waist circumference was measured to the nearest 0.1 cm; waist circumference was measured at the midpoint between the base of the rib case and the upper part of the border of the iliac crest. Waist–height ratio was calculated as waist circumference (cm) divided by height (cm) a measure of central adiposity.

Blood pressure was measured after 10 min of supine rest using a size-adjusted arm cuff. Three measurements were taken, and the mean of the two later was calculated. Blood pressure *z*-scores were obtained from a large American material [17]. MAP was calculated as MAP = diastolic blood pressure + 1/3 (systolic blood pressure − diastolic blood pressure).

Ethnicity was collected using self-identification by the participants and their parents.

Fasting blood samples were taken, and HbA1c and lipids (total, LDL, high-density lipoprotein [HDL]) cholesterol, and triglycerides [TG]) were measured using standardized methods.

#### 2.1.1. Arterial Stiffness

CfPWV was measured using the Sphygmocor XCEL system (Atcor Medical, Sydney, Australia) after 10 min of supine rest. The measurements were performed in the morning (between 8:00 and 10:00 am) in a quiet, temperature-stable (20–22°C) room. Distance from the carotid artery to the cuff was measured using a caliper. The examination was performed according to guidelines using the direct method [7]. Two measurements were performed, and if they differed with more than 0.5 m/sa third was taken. The average of the two measurements (closest to each other) were used for analysis. The reference material from Reusz' et al. [18] was used to compute cfPWV *z*-scores. Arterial stiffness was defined as elevated if cfPWV was equal to or above the 90th percentile of the age and sex-based reference material [19].

#### 2.1.2. Regular Screening for Complications

A Biothesiometer (Kjaerulff, Denmark), examining vibration sensation as a measure of sensory neuropathy, was performed on the right big toe twice, and the average was calculated. Morning urine samples from 3 days were analyzed for albumin–creatinine ratio by an enzyme immunoassay, and the geometric mean was calculated for each participant.

Retinopathy screening was performed using the OPTOS device (Nikon, OPTOS Monaco, P200TE, UK). An ultra-widefield retinal image was performed for each eye. The pictures were graded according to current guidelines [20] by a trained ophthalmologist.

#### 2.1.3. FibroScan

While the participant was still fasting, a FibroScan compact 530 (Echosens, Paris, France) was performed after 5 min of rest. The M-probe was used for all, except when the XL-probe was recommended by the FibroScan due to the depth of the liver. All examinations were performed by specially trained clinical staff.

Liver stiffness was measured in kilopascal (kPa). According to guidelines [21], an examination was approved if 10 valid measurements had an interquartile range of less than 30% of the mean of liver stiffness. Measurement by the FibroScan was missing, by random, for 32 participants.

#### 2.1.4. Metabolic Outcome

Information on insulin treatment, including type and administration and data from the continuous glucose monitor (CGM), was collected for the latest 14 days. Time-in-range was defined as the percentage of time spent in the target glucose range (glucose between 3.9 and 10 mmol/l).

Insulin sensitivity was calculated using a surrogate marker: log insulin sensitivity = 4.64725 − 0.02032 × (waist circumference; cm)−0.09779 × (HbA1c; %) − 0.00235 × (TG; mg/dl) [22].

Information on diabetes onset and retrospective HbA1c measures were obtained from the medical records. A retrospective HbA1c was calculated as the mean of two measurements per year, if available. The first 12 months after diagnosis were excluded due to possible remission phase. The HbA1c measure nearest to the birthday of each participant was selected, and the other was separated by at least 90 days to avoid interaction.

Comparisons between sexes were performed using unpaired sample *t*-test for normal distributed and the Mann–Whitney for nonnormally distributed variables.

#### 2.1.5. Statistics

Normally distributed continuous variables are presented as mean with standard deviation (SD) and nonnormally distributed variables as median with interquartile range. Comparisons between sexes were performed using unpaired sample *t*-test for normal. The comparisons between sexes were chosen due to the higher risk of cardiovascular disease in general in males. Categorial variables are summarized as numbers with percentages and the *X*^2^ test was applied for comparison between groups.

Unadjusted linear regression models were applied to determine the crude association between cfPWV and other risk factors. Next, adjusted linear regression models, including age, sex, diabetes duration, time-in-range, retrospective HbA1c, BMI *z*-score, LDL-cholesterol, and MAP, were applied. Standardized regression coefficients (*β*) with 95% confidence intervals (CIs) are reported.

Sensitivity analysis using current HbA1c rather than retrospective HbA1c was performed to test the robustness of the model. A second sensitivity analysis was performed, including the mode of insulin delivery to the adjusted model.

Model assumptions were ascertained in all analyses. Two-sided *p*-value <0.05 was considered statistically significant. All statistical analyses were performed with R studio, R version 4.2.2.

#### 2.1.6. Ethics

Written informed consent was collected from both parents and legal guardians. According to Danish law participants at the age of 15 years or older also gave written informed consent. If the participant was aged 18 years old, they only gave written informed consent themself.

The study was conducted according to the Helsinki Declaration and was approved by the Danish Ethics Committee of the Capital Region (H-20038547). The study was registered at ClinicalTrias.gov (NCT05159856).

## 3. Results

### 3.1. Descriptive Analysis

Clinical characteristics for the analyzed population and stratified by sex are presented in Table 1. The 125 children and adolescents included had a median (interquartile range) age of 14.2 years (11.9–16.3) and diabetes duration of 4.7 years (2.6–8.5), and 66 (53%) were male. The majority were Scandinavian and non-Hispanic white (87.5%). The median (interquartile range) of the current HbA1c level was 7.0% (6.5–7.9), (53 mmol/mol: 48–63), and time-in-range 63% (54–75).

Characteristics were comparable between males and females, except for BMI *z*-score and waist-height ratio, which both were significantly higher for females (*p*=0.006 and *p*=0.034, respectively) and liver stiffness which was significantly higher for males (*p*=0.002).

Three reported smoking at social gatherings/parties. The large majority were treated with CSII (82%). All except two used CGM (98.5%). These two were excluded from all analyses, including time-in-range.

All participants included in the analyses had normal fundus photos. Biothesiometry, as a measure of neuropathy, was within the normal range for all participants, and all had normoalbuminuria (<30 mg/g).

### 3.2. Prevalence of Increased cfPWV and Associated Clinical Characteristics

**Figure 1:**
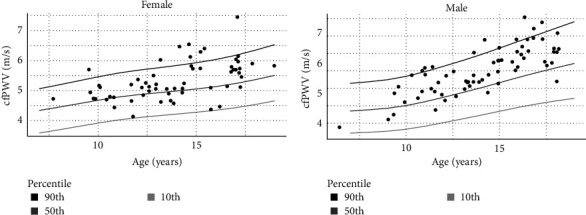
Distribution of arterial stiffness measured by cfPWV. Distribution of cfPWV (m/s) for (A) females and (B) males. The percentiles are based on reference material based on healthy children and adolescents from Reusz et al. [18]. cfPWV, carotid–femoral pulse-wave-velocity.

The prevalence of increased arterial stiffness (cfPWV *z*-score over the 90th percentile) was 16%. The majority (81%) of the participants had a cfPWV above the 50th percentile (Figure 1).

Unadjusted analyses comparing the group with increased cfPWV (*n* = 20) to the group within the normal range (*n* = 105) demonstrated that the participants with increased cfPWV were characterized by lower time-in-range (*p*=0.011) and LDL cholesterol (*p*=0.026), all other characteristics were comparable.

Unadjusted linear regression analyses demonstrated that higher cfPWV (as a continuous variable) was associated with longer diabetes duration, higher age, HbA1c (current and retrospective), MAP and liver stiffness, and lower time-in-range and lower insulin sensitivity (Table 2).

Multivariate linear regression analysis, including age, sex, diabetes duration, time-in-range, retrospective HbA1c, BMI *z*-score, LDL-cholesterol, and MAP, demonstrated a significant association between higher cfPWV and higher age (*p*  < 0.001) and lower time-in-range (*p*=0.007). No other associations were found (Table 3).

A sensitivity analysis replacing retrospective HbA1c with current HbA1c in the adjusted regression model showed consistent results, with no association between cfPWV and HbA1c (*p*=0.579).

The cfPWV was not associated with the insulin delivery system (CSII [*n* = 102] vs. multiple daily injections [*n* = 23]) (unadjusted *p*=0.114), and inclusion of insulin delivery system to the adjusted model did not change the associations described above.

The association between cfPWV and insulin sensitivity was tested in a separate model, as the calculation of insulin sensitivity is based on HbA1c, TG, and abdominal circumference. There was no association between cfPWV and insulin sensitivity after adjustment for age, sex, diabetes duration, and MAP (*p*=0.102).

As information on liver stiffness was missing in 32 (25%) of the participants, the association between cfPWV and liver stiffness was tested in a separate model. No association was demonstrated in the adjusted (*p*=0.219) analysis.

#### 3.2.1. Subgroup Analysis

An explorative analysis comparing the 13 participants with the 10% highest and the 13 with the 10% lowest cfPWV *z*-score showed that participants in the “high cfPWV group” were characterized by significantly longer diabetes duration, higher MAP and lower time-in-range and insulin sensitivity than the “low cfPWV group.”

## 4. Discussion

In this study, we investigated the prevalence of elevated arterial stiffness (>90th percentile) in a European cohort of children and adolescents with type 1 diabetes under modern technology-based care. The prevalence was 16%, which is comparable to earlier findings of 10% [19] and 12% [23] for elevated cfPWV, both using the 90th percentile as a cutoff but compared to their own control group. As our cohort was younger, had a shorter duration of type 1 diabetes, better glycemic control, and almost no classic long-term diabetes complications compared to the earlier larger cohorts from the SEARCH study [19, 23], the prevalence was unexpectedly high. Moreover, 81% of the participants in our cohort had cfPWV above the 50th percentile of a reference based on data from a normal population (adjusted for age and sex) [18]. This underscores that despite advancements in technology and improved overall metabolic outcomes, children and adolescents with type 1 diabetes still present with a high prevalence of elevated arterial stiffness. The 90th percentile has been stated [6, 19] as the cutoff for abnormally elevated arterial stiffness, an early sign of atherosclerosis, indicating that 16% of children and adolescents with type 1 diabetes are at increased risk of cardiovascular disease later in life. This finding underscores the importance of vigilance in managing cardiovascular health in this population subgroup.

Furthermore, we explored the association between arterial stiffness and established as well as potential novel risk factors. This study is the first to demonstrate an independent association between higher arterial stiffness and lower time-in-range. Intriguingly, no association between arterial stiffness and HbA1c was found, whether based on retrospective information or current level. Previous cross-sectional studies have, in accordance with our findings, not demonstrated any association between cfPWV and HbA1c in children and adolescents with type 1 diabetes [1, 12, 19]. This could be due to HbA1c being a more diverse measure as it may be an expression of diverse glucose patterns, whereas time-in-range is a more sensitive measure of the glucose level [24]. While a cross-sectional study of 54 adults with type 1 diabetes found no significant association between arterial stiffness and time-in-range, it did reveal a correlation with the mean HbA1c levels over the past 10-year period [25]. Interestingly, two large studies, including adults with type 2 diabetes, have also shown an association between increased arterial stiffness and lower time-in-range [26, 27], but this association has never been investigated in children with type 1 diabetes before.

We expected a positive association between cfPWV and MAP, as previously shown [1], but the association was only present in crude analyses and not after adjustment for other cardiovascular risk factors. Likewise, we could not demonstrate an association between cfPWV and central obesity or lipids, as seen in previous studies [10, 12].

Few studies have investigated treatment options for increased arterial stiffness in children and adolescents with type 1 diabetes. A randomized controlled trial investigated whether motivational interviewing added to standard educational care at each clinical visit for 12 months would improve vascular health (including cfPWV) in 47 adolescents with poorly controlled type 1 diabetes [28]. No effect of adding motivational interviewing to standard care was found on neither cfPWV nor HbA1c.

A randomized trial, including 14 participants in each of three treatment groups, demonstrated that treatment with empagliflozin and metformin in combination for 12 weeks improved arterial stiffness significantly when compared to metformin treatment alone in adults with type 1 diabetes [29]. The combination treatment also lowered HbA1c significantly over 12-weeks compared to baseline. Empagliflozin treatment alone also improved arterial stiffness compared to baseline but to a decreased extent compared to the combination treatment and did not lower HbA1c significantly [29].

More larger intervention studies are needed. Hence, the most effective strategy at present continues to emphasize the importance of mitigating arterial stiffness through the management of cardiovascular risk factors, notably including glycemic control, as evidenced by the findings of this study [10, 12].

### 4.1. Strengths and Limitations

This study presents a thoroughly examined and well-characterized cohort. Arterial stiffness was assessed using the gold standard method. A limitation is the lack of a nondiabetic control group; we, therefore, applied a large reference material and converted it to *z*-scores.

The lack of information on physical activity and aerobic fitness is a limitation, as high levels of these factors have benefits for cardiovascular health. We used BMI *z*-score as an objective measure for body composition, as a DXA scan or MRI was not available. Most of the participants were Scandinavian and non-Hispanic white, limiting the generalization of the findings to other ethnicities.

The majority of the participants used CGM, which may restrict the extension of the study's findings to individuals with CGM.

Moreover, the cross-sectional nature prevents the evaluation of causality. Future follow-up of the cohort will enable causality analyses and a deeper understanding of the mechanisms affecting arterial stiffness.

## 5. Conclusion

In conclusion, this study indicates that despite modern technology and better overall metabolic outcomes, children and adolescents with type 1 diabetes still present with a high prevalence of arterial stiffness, an established early sign of atherosclerosis. Higher arterial stiffness was associated with higher age and lower time-in-range, independent of HbA1c, demonstrating the importance of tight blood glucose control.

## Figures and Tables

**Table 1:** Clinical characteristics in the total population and stratified by sex.

Clinical variables	Total (*n* = 125)	Male (*n* = 66)	Female (*n* = 59)	*p*
Age (years)	14.2 (11.90, 16.3)	14.4 (11.8, 16.3)	14.0 (12.2, 16.6)	0.669
Diabetes duration (years)	4.7 (2.7, 8.4)	4.3 (2.4, 7.0)	5.5 (3.3, 8.6)	0.207
BMI *z*-score	0.5 ± 1.0	0.3 ± 1.1	0.8 ± 1.0	0.006
Waist–height ratio	0.43 (0.41, 0.47)	0.43 (0.41, 0.46)	0.45 (0.42, 0.48)	0.034
Systolic blood pressure (mmHg)	106 ± 7	107 ± 7	105 ± 8	0.121
Diastolic blood pressure (mmHg)	57 ± 4	57 ± 4	57 ± 4	0.455
MAP (mmHg)	73 ± 5	74 ± 4	73 ± 5	0.198
cfPWV (m/s)	5.4 ± 0.7	5.5 ± 0.7	5.3 ± 0.6	0.121
Time-in-range (%)	63 (53, 75)	65 (58, 75)	61 (51, 73)	0.133
Current HbA1c (%)	7.0 (6.5, 7.9)	7.0 (6.5, 7.6)	7.1 (6.7, 8.1)	0.303
Current HbA1c (mmol/mol)	53 (48, 63)	53.0 (48, 60)	54 (50, 65)	0.303
Retrospective HbA1c (%)	7.3 (6.5, 7.9)	7.2 (6.6, 7.6)	7.3 (6.7, 7.8)	0.319
Retrospective HbA1c (mmol/mol)	56 (48, 63)	55 (49, 60)	56 (50, 62)	0.319
LDL cholesterol (mmol/l)	2.2 ± 0.5	2.1 ± 0.5	2.2 ± 0.5	0.103
HDL cholesterol (mmol/l)	1.5 ± 0.3	1.5 ± 0.4	1.5 ± 0.3	0.763
Liver stiffness (kPa)	4.4 (3.8, 5.2)	4.7 (4.2, 5.5)	4.0 (3.5, 4.9)	0.002
Total daily insulin dose (IU)	45.7 (35.6, 62.1)	49.3 ± 17.8	48.4 ± 20.0	0.771
CSII users	102 (82%)	52 (79%)	51 (85%)	0.531
Insulin sensitivity score	10.17 ± 2.66	10.37 ± 2.73	9.93 ± 2.57	0.373

*Note:* Data represent mean ± standard deviation or median (interquartile range) or *n* (%). *p* for the difference between sexes was calculated using an unpaired sample *t*-test for normally distributed and the Mann–Whitney test for non-normally distributed variables. The *χ*^2^-test was used for the categorical variable. The insulin sensitivity score was calculated as a surrogate marker, as described in Section 2.

Abbreviations: BMI, body mass index; cfPWV, carotid–femoral pulse-wave velocity; CSII, continuous subcutaneous insulin infusion; HbA1c, hemoglobin A1c; HDL, high-density lipoprotein; IU, insulin units; LDL, low-density lipoprotein; MAP, mean arterial pressure.

**Table 2:** Univariate association between cfPWV and clinical variables.

Clinical variables	*β*	(95% CI)	*p*
Age	0.44	(0.35, 0.52)	<0.001
Sex (female)	−0.18	(−0.41, 0.04)	0.121
Diabetes duration	0.14	(0.02, 0.25)	0.019
Current HbA1c	0.20	(0.09, 0.31)	<0.001
Time-in-range	−0.27	(−0.38, −0.16)	<0.001
BMI *z*-score	0.01	(−0.11, 0.12)	0.943
Retrospective HbA1c	0.22	(0.11, 0.3)	<0.001
Waist–height ratio	0.02	(−0.09, 0.14)	0.676
LDL cholesterol	−0.05	(−0.16, 0.07)	0.414
MAP	0.19	(0.08, 0.31)	<0.001
Insulin sensitivity	−0.33	(−0.43, −0.22)	<0.001
Liver stiffness	0.17	(0.05, 0.29)	0.007

*Note:* Associations were calculated using unadjusted linear regression and are reported as standardized beta coefficients with 95% confidence intervals (95% CI). The insulin sensitivity score was calculated as a surrogate marker, as described in Section 2.

Abbreviations: BMI, body mass index; cfPWV, carotid–femoral pulse-wave velocity; HbA1c, hemoglobin A1c; LDL, low-density lipoprotein; MAP, mean arterial pressure.

**Table 3:** Multivariate linear regression model for the association to cfPWV.

Clinical variables	*β*	(95% CI)	*p*
Age	0.37	(0.27, 0.48)	<0.001
Sex (female)	−0.13	(−0.31, 0.06)	0.168
Diabetes duration	0.02	(−0.07, 0.11)	0.736
BMI *z*-score	−0.06	(−0.15, 0.04)	0.257
MAP	0.08	(−0.02, 0.17)	0.129
Time-in-range	−0.15	(−027, −0.04)	0.007
Retrospective HbA1c	0.01	(−0.12, 0.11)	0.958
LDL cholesterol	−0.04	(−0.14, 0.05)	0.373

*Note:* Associations were calculated using adjusted linear regression and are reported as standardized beta coefficients with 95% confidence intervals (95% CI). *R*-squared (*R*^2^) for the total model was 0.49.

Abbreviations: BMI, body mass index; cfPWV, carotid–femoral pulse-wave velocity; HbA1c, hemoglobin A1c; LDL, low-density lipoprotein; MAP, mean arterial pressure.

## Data Availability

Data are available from the corresponding author upon reasonable request.
